# Cross-Cultural Adaptation and Psychometric Testing of the Portuguese Version of the Iceland-Family Illness Beliefs Questionnaire

**DOI:** 10.1177/10748407241226955

**Published:** 2024-01-29

**Authors:** Sara Lemos, Luísa Andrade, Lígia Lima, Teresa Martins, Erla Kolbrún Svavarsdottir, Maria Do Céu Barbieri-Figueiredo

**Affiliations:** 1 Institute of Biomedical Sciences Abel Salazar, University of Porto, Portugal; 2 Center for Health Technology and Services Research, Portugal; 3 Center for Health Technology and Services Research at the Health Research Network, Portugal; 4 Nursing School of Porto, Portugal; 5 University of Iceland, Reykjavik, Iceland; 6 Landspitali University Hospital, Reykjavik, Iceland; 7 Nursing Department, University of Huelva, Spain

**Keywords:** illness beliefs, chronic illness, children, adolescent, family nursing, psychometrics

## Abstract

Illness beliefs have a role in the adaptation, coping, well-being, healing, and recovery in families of children/adolescents with chronic illness. The assessment of family illness beliefs can support family nursing interventions that address the suffering of family members when illness arises. The purpose of this study was to translate, cross-culturally adapt, and psychometrically test the Portuguese version of the Iceland-Family Illness Beliefs Questionnaire. A sample of 237 parents of children/adolescents who experienced chronic health conditions completed the online questionnaire. The original factor model was tested through confirmatory factorial analysis. The results showed satisfactory model fit indices (χ^2^/gl = 3.004; comparative fit index [CFI] = 0.90; root mean square error of approximation [RMSEA] = 0.092) and internal consistency (Cronbach’s α = 0.74). The instrument showed good psychometric characteristics of validity and reliability, suggesting it may be useful in the assessment of illness beliefs in families experiencing a pediatric chronic illness.

## Background

The prevalence of pediatric chronic disease has increased in recent decades (Gallo et al., 2021). It is estimated that 10% to 20% of all children and adolescents are affected by an illness that lasts longer than 3 months and may persist or have recurrent episodes (Knafl et al., 2017; Van Cleave et al., 2010). The complexity of health care needs and the treatment and management of pediatric chronic illness have psychological, behavioral, and social repercussions on all family members (Fairfax et al., 2019). Undoubtedly, pediatric chronic illness is one of the most traumatic and challenging events for the family unit, leading to various ways of coping with the illness (Knafl et al., 2017; Lummer-Aikey & Goldstein, 2021).

In more severe and complex health conditions, families have their dreams, expectations, values, behaviors, and beliefs regarding life and health threatened. The way in which the family unit responds to the diagnosis and prognosis of illness is influenced by their individual and family beliefs (Wright & Bell, 2021). In this context, approaching the family from a systemic perspective is particularly useful, as it attends to family identity, individual characteristics, and family dynamics (Galvin et al., 2019; Shajani & Snell, 2023).

Wright and Bell (2021) define beliefs as “the lens through which we view the world, guide the choices we make, the behaviors we demonstrate, and our related feelings” (p. 14). Beliefs are shaped and substantially shifted through our interactions with others and ourselves in harmony with the condition that we live in (Wright & Bell, 2021). In pediatric chronic diseases, the beliefs about the illness held by the individual, his or her family members, and the health professionals themselves influence the way in which families deal with the experience of the illness. Hence, it is important that nurses, and all health professionals in general, listen carefully to understand the family’s beliefs, because it is not necessarily the clinical situation that predicts family suffering, but the beliefs that family members hold about the illness (Wright & Bell, 2021).

The Illness Beliefs Model (IBM) offers a theoretical framework developed from research conducted at the Family Nursing Unit at the University of Calgary, Canada. This theoretical model recognizes that beliefs about illness held by individuals, their families, and health care professionals influence relationships and behavior in illness situations, as well as suffering and healing. To understand illness beliefs, it is important to know the relational, social, cultural, and religious context in which individuals and their families live. The IBM identifies “core” beliefs, those that are at the core of suffering and healing, highlighting beliefs about the etiology, diagnosis, prognosis, control, and influence of the illness on the individual and family, and the support received by health professionals (Bell & Wright, 2015; Wright & Bell, 2021).

Illness beliefs can be facilitating or constraining. Facilitating beliefs are those that are helpful or useful to find solutions in a given context, whereas constraining beliefs are those that hinder or decrease the possibilities of finding solutions or solving problems and increase suffering. From this perspective, it is important that health professionals assess the beliefs of individuals and families to explore, challenge, and change constraining beliefs and recognize, reinforce, and strengthen facilitating beliefs. When using the IBM model in clinical settings, nurses identify the beliefs that influence the coping process and establish interventions aimed at managing and lessening the family’s experiences of suffering (Arestedt et al., 2015; Bell & Wright, 2015).

The influence of illness beliefs of families with a child experiencing chronic illness and the process of adaptation to the illness have been described. Research by Sato and colleagues (2015) deepened understanding about beliefs and experiences of mothers of children with disabilities in the Japanese sociocultural context. They identified beliefs about relationships, beliefs about death and illness, beliefs about norms, and beliefs about self in their Common Tentative Framework of Japanese Family Beliefs. Wacharasin and colleagues (2015), in Thailand, developed a program based on the IBM and reported that family caregivers found

that the Family Empowerment Program (FEP) helped them share beliefs and experiences related to caring for their child with thalassemia, make decisions related to families’ problems/needs and beliefs, provide each other mutual social support, and develop increased ability to manage care for their chronically ill child through sharing information and learning from other family caregivers about family functioning, family management, and family relationships (p. 295).

Pediatric chronic disease is often complex, and families endure a major dependence on health care services and practitioners, requiring a process of adjustment from the entire family unit. It is of utmost importance for health care providers to identify and assess family illness beliefs, which are essential for the development of interventions aimed to promote the process of family’s adaptation to the disease, and reduce illness suffering and promote family healing (Bell & Wright, 2015).

This research contributes to family nursing research and practice, by developing a Portuguese version of the Iceland-Family Illness Beliefs Questionnaire (ICE-FIBQ) by Gisladottir and Svavarsdottir (2016), an instrument that assesses family illness beliefs based on the conceptual foundation of the IBM (Wright & Bell, 2021). The development of the Portuguese version involved the cross-cultural adaptation of the Icelandic original version and the validation of the European Portuguese version.

## Method

The aims of this study were to cross-culturally adapt the ICE-FIBQ to a Portuguese population and examine the psychometric properties of the Portuguese version of the instrument.

This research was part of an international study-program, “Integrating Pediatric Chronic Illnesses and/or Disorders into Family Life: An International Longitudinal Study” coordinated by a team from the University of Iceland with researchers from Denmark, Finland, Portugal, and Spain.

### Study Design and Participants

This was a methodological study. The sample was recruited in a pediatric day hospital and outpatient clinics from four hospitals in Northern Portugal, between May 2021 and January 2022. Participants were asked to respond to an online survey using REDCap Software (Research Electronic Data Capture) data capture tools (Harris et al., 2019). A convenience sample was obtained with parents and/or caregivers of children/adolescents with a medical diagnosis of one or more chronic health conditions. Inclusion criteria were (a) a medical diagnosis of more than 6 months, (b) participants’ proficiency in the Portuguese language, and (c) with access to an electronic device with internet. In total, 507 participants were invited to participate in the study; of which, 237 parents/caregivers completed all instrument items and were included in the analysis (a participation rate of 46.75%). Demographic data included the parent’s age, gender, educational level, marital status, and employment status, as well as children-/adolescent-related variables that included, gender, age, diagnosis, and duration of the illness.

### Iceland-Family Illness Beliefs Questionnaire

The ICE-FIBQ is a seven-item questionnaire developed in Iceland by Gisladottir and Svavarsdottir (2016) that measures individual family members’ illness beliefs. Each item is scored on a 5-point *Likert-type* scale, where 1, 2, 3, 4, and 5 correspond to *never*, *almost never*, *sometimes*, *most of the time*, and *all of the time*, respectively. The score total ranges from 7 to 35 points. A higher score reports confidence in the subjective “truth” of a family member’s illness beliefs, meaning that illness beliefs are present in the process of coping with illness. According to the theoretical basis of the IBM, the seven items of this instrument assess the illness beliefs about (a) cause of illness, (b) control of illness on family and control of family on illness, (c) effect of illness on the individual and family, (d) illness suffering, and (e) support received from health care professionals during illness. The original ICE-FIBQ showed good internal consistency with a Cronbach’s alpha of 0.79 (Gisladottir & Svavarsdottir, 2016).

### Cross-Cultural Adaptation Process of the Instrument

**Figure 1.**
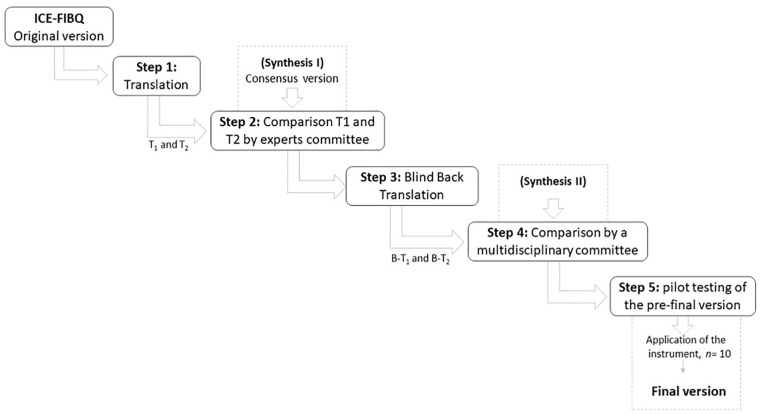
Flowchart of Translation and Cross-Cultural Adaptation Process of ICE-FIBQ Into Portuguese. *Note.* ICE-FIBQ = Iceland-Family Illness Beliefs Questionnaire.

A multistage procedure was followed for the process of cross-cultural adaptation, as recommended by Beaton et al. (2000), described in Figure 1. This process was developed in five steps. In Step I, the translation of the original instrument into the language of the target group was started. The translation of the original instrument from English into European Portuguese was performed by two independent bilingual translators (T), both native speakers of Portuguese. One of the translators (T1) had previous knowledge about pediatric nursing and family nursing and its terminology, which was the content area of the construct of the original instrument. The second translator (T2) had knowledge of the cultural and linguistic nuances of the English language. As a result of this step, two translated versions were obtained. In Step II, the research team compared and combined the two translations into a single version. In this step, all members of research team identified ambiguities and discrepancies of words, sentences, and meanings. With the arguments of T1 and T2, a consensus version was developed. The first version of the Portuguese version of the scale was a result of this work (Synthesis I). In Step III, a back-translation of the first version was done by two new bilingual translators (B-T1 and B-T2). They were native English speakers, fluent in Portuguese language, without any previous knowledge of the original instrument. The objective of this step was to ascertain the consistency of the translations performed in the first step of this process. In Step IV, a research team meeting was held to create the prefinal version (Synthesis II). The objective of this step was to discuss and resolve the adequacy of the translation and back-translation to determine whether the semantic, idiomatic, cultural, and conceptual equivalences had been maintained. Finally, Step V was a pretest, which included 10 participants, who answered the prefinal version of the questionnaire. The purpose of this last step was to assess the acceptability and comprehensibility of the questionnaire by the participants from the target population. Through a semi-structured questionnaire, we discussed with the participants their understanding of each item and their answers, the relevance of the item, and the clarity of the instructions. The pretest did not result in the need to reformulate the content of the scale, as most parents reported that the items were clear and easy to read. As a result of this last step, a final Portuguese version was created.

### Ethical Considerations

This study was conducted in accordance with the Declaration of Helsinki. It was approved by the four Ethics Committees of the Hospital Institutions of Northern Portugal involved in this study (No. 52/2021, No. 38/2021, No. 98_2021, and No. 16/2021). All participants were provided with information that the survey was voluntary, confidential, and anonymous. After the participants signed an informed consent form, they were provided an e-mail contact to receive the link to access the online questionnaire in the REDCap Software program.

## Data Analysis

Statistical analysis used IBM SPSS Statistics, version 28 and IBM AMOS, version 27 software for Windows. Internal consistency was evaluated by Cronbach’s alpha coefficient; alpha values above 0.70 were considered acceptable (Polit & Yang, 2016). The test–retest was calculated using the intraclass correlation coefficient; satisfactory stability was considered if values were above 0.70 (Polit & Yang, 2016).

Structural equation analysis was performed through confirmatory factor analysis (CFA) to test the unifactorial model of the ICE-FIBQ. The covariance matrix was considered as input using the maximum likelihood estimation method. The existence of outliers was evaluated by the square of the Mahalanobis distance, and normality was calculated through the coefficient of asymmetry and multivariate kurtosis. The quality of the fit of the model was performed in accordance with the indices and respective reference values (Kline, 2016; Marôco, 2021). The quality of the local fit was assessed by factor weights and the individual reliability of the items. The comparative fit index (CFI) and the root mean square error approximation (RMSEA), with its confidence intervals (CIs) and modification indexes were also considered.

## Results

In total, 237 parents participated in this study. Table 1 summarizes the demographic characteristics of the parents and their children. Participants included 207 mothers (87.3%) and 28 fathers (11.8%). More than half of them were in the age group of 41 to 50 years (56.1%) and were educated at secondary level and university degree (37.6% and 32.9%, respectively). About the children/adolescent, the sample comprised a similar number of female and male (51.1% and 48.1%, respectively) and the most predominant age group range was 11 to 18 years (53.5%). Some children/adolescents have been diagnosed with more than one diagnosis of disease and/or chronic disorder. Of these diagnoses, 244 were of a chronic disease, most frequently a disease of respiratory system (16.3%) and gastrointestinal system (12.3%); 50 diagnoses were of a psychological disorder (16.7%).

**Table 1.** Descriptive Characteristics of the Sample (n = 237).

Demographic characteristics	*N*	%
Participants		
Gender		
Male	28	11.8
Female	207	87.3
No answer	2	0.8
Age (years)		
18 to 30	8	3.4
31 to 40	73	30.8
41 to 50	133	56.1
51 to 60	20	8.4
≥61	3	1.3
Education level		
Elementary school	37	15.6
Secondary education	89	37.6
Professional course	14	5.9
University degree	78	32.9
Other	19	8.0
Marital status		
Married/cohabiting	194	81.9
Single/divorced	36	15.2
Widowed	6	2.5
No answer	1	0.4
Employment status		
Full-time	136	57.4
Part-time	19	8.0
Unemployed	27	11.4
Full-time and second employ	12	5.2
Other	43	18.0
Children/adolescents		
Gender		
Female	114	48.1
Male	121	51.1
No answer	2	0.8
Age (years)		
1–3	33	14.0
4–6	24	10.1
7–10	53	22.4
11–18	127	53.5
Diagnoses of chronic condition (N=300)		
Chronic illness	244	81.3
Developmental disorder	6	2.0
Psychological disorder	50	16.7

The distribution of participants’ responses to the Portuguese version of the ICE-FIBQ is shown in Table 2. The scale showed good acceptability, with missing data between 0.4% and 3.8%. By analyzing the distribution of answers, it is possible to observe that the answers were distributed over all the options of the scale, with a more concentrated distribution at the higher levels of the scale.

**Table 2.** Response Frequencies to the Items of the ICE-FIBQ (n = 237).

Item	Results, *n* (%)					Missing, *n* (%)
	*Never*	*Almost never*	*Sometimes*	*Most of the time*	*All of the time*	
B1 . . . I know what is the cause of the chronic illness/disorder that we are now dealing with	42 (17.7)	16 (6.8)	36 (15.2)	51 (21.5)	91 (38.4)	1 (0.4)
B2 . . . I know how much control my family has over the chronic illness/disorder	11 (4.6)	14 (5.9)	73 (30.8)	72 (30.4)	67 (28.3)	0
B3 . . . I know how much control the chronic illness/disorder has over my family	13 (5.5)	17 (7.2)	67 (28.3)	72 (30.4)	66 (27.8)	2 (0.8)
B4 . . . I would know what the effect (if any) would be on the chronic illness/disorder if my partner and I would agree on handling the situation at home	15 (6.3)	23 (9.7)	49 (20.7)	56 (23.6)	85 (35.9)	9 (3.8)
B5 . . . I know who is suffering the most or has the most difficulty in our family, because of the changes in our family life due to the chronic illness/disorder	8 (3.4)	8 (3.4)	35 (14.8)	68 (28.7)	116 (48.9)	2 (0.8)
B6 . . . I know what has been the most useful thing health professionals have offered to help me to cope with my suffering regarding our chronic illness/disorder	8 (3.4)	17 (7.2)	50 (21.1)	80 (33.8)	79 (33.3)	3 (1.3)
B7 . . . I know what has been the least useful thing health professionals have offered to help me to cope with my suffering regarding our chronic illness/disorder	25 (10.5)	34 (14.3)	57 (24.1)	56 (23.6)	62 (26.2)	3 (1.3)

*Note.* ICE-FIBQ = Iceland-Family Illness Beliefs Questionnaire.

The items’ mean scores were also calculated and, as shown in Table 3, were all above the scale average, ranging from 3.41 to 4.17.

**Table 3.** Descriptive Statistics of the Items on the ICE-FIBQ (n = 237).

Item	Min–max	*M* (*SD*)	Md	Mo	Skew	K
B1 . . . I know what is the cause of the chronic illness/disorder that we are now dealing with	1–5	3.56 (1.49)	4.00	5	−0.646	−1.017
B2 . . . I know how much control my family has over the chronic illness/disorder	1–5	3.72 (1.08)	4.00	3	−0.593	−0.108
B3 . . . I know how much control the chronic illness/disorder has over my family	1–5	3.69 (1.12)	4.00	4	−0.617	−0.204
B4 . . . I would know what the effect (if any) would be on the chronic illness/disorder if my partner and I would agree on handling the situation at home	1–5	3.76 (1.24)	4.00	5	−0.699	−0.520
B5 . . . I know who is suffering the most or has the most difficulty in our family, because of the changes in our family life due to the chronic illness/disorder	1–5	4.17 (1.03)	4.00	5	−1.305	1.306
B6 . . . I know what has been the most useful thing health professionals have offered to help me to cope with my suffering regarding our chronic illness/disorder	1–5	3.88 (1.07)	4.00	4	−0.797	0.061
B7 . . . I know what has been the least useful thing health professionals have offered to help me to cope with my suffering regarding our chronic illness/disorder	1–5	3.41 (1.31)	3.00	5	−0.365	−0.959
Total score	0.71–5	3.70 (0.80)	3.71	5	−0.263	0.164

*Note.* ICE-FIBQ = Iceland-Family Illness Beliefs Questionnaire.

### Reliability and Temporal Stability

Internal consistency was satisfactory (0.74) for the Portuguese version of the ICE-FIBQ scale. There were 34 participants (14.4%) who completed the instrument twice, approximately 4 weeks following the first administration. The results of the test–retest analysis (ICC) showed a satisfactory stability over time (0.71; see Table 4).

**Table 4.** ICC (95% CI) for the Test–Retest Group (n = 34).

ICC^a^ (95% CI)			
	ICC^b,c^	95% CI	
		Lower bound	Upper bound
ICE-FIBQ	0.71	0.43	0.85

*Note.* Two-way random effects model where people are random and measure effects are fixed. ICC = intraclass correlation; CI = confidence interval; ICE-FIBQ = Iceland-Family Illness Beliefs Questionnaire.

a Type A intraclass correlation coefficients using an absolute agreement definition. ^b^ This estimate is computed assuming the interaction effect is absent, because it is not estimable otherwise. ^c^ Average measures.

### Construct Validity—Confirmatory Factor Analysis

**Figure 2.**
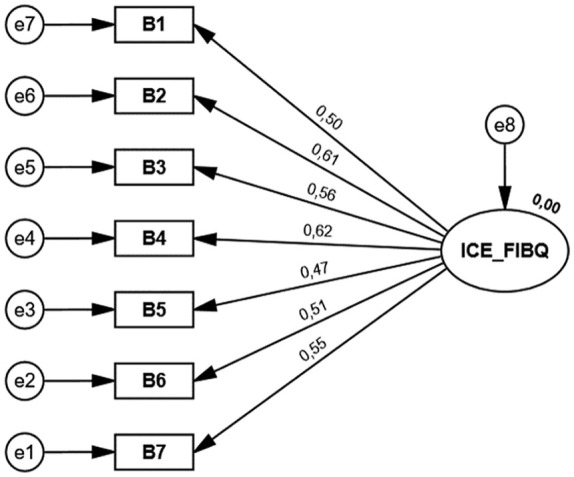
Confirmatory Factor Analysis of the European Portuguese Version of Iceland-Family Illness Beliefs Questionnaire.

Figure 2 shows the model tested and that was based on the factorial structure proposed by the original authors of the ICE-FIBQ scale (Gisladottir & Svavarsdottir, 2016). According to the one-factor solution model, the CFA showed an acceptable fit with the following results: χ^2^/*df* = 3.004, CFI = 0.902, PCFI = 0.601, and RMSEA = 0.092.

## Discussion

The present study describes the translation and cross-cultural adaptation of the ICE-FIBQ scale into European Portuguese. The translation and cultural adaptation process obtained positive feedback from parents/caregivers of children/adolescents with a chronic condition. As a second aim, we assessed the psychometric properties of the Portuguese version of the ICE-FIBQ scale to ensure the validity and reliability of the instrument and obtained promising results.

To our knowledge, this is the first time the instrument was used in a pediatric context in Portugal to assess family member’s illness beliefs. Our findings showed that the parents of children/adolescents with chronic health conditions were able and willing to offer their subjective “truth” of their illness beliefs. The items’ means were overall higher than the ones found in the original version of the instrument probably due to cultural reasons or due to the characteristics of the samples in each study (Gisladottir & Svavarsdottir, 2016). From an analysis of psychometric properties of the items, our results showed a distribution of responses across all response categories of the scale, with a greater concentration on the higher values of the Likert-type scale. Nevertheless, the items do not show ceiling or floor effects. Also, we verified that the item, “I would know what the effect (if any) would be on the chronic illness/disorder if my partner and I would agree on handling the situation at home” had more missing values compared with the other items. This finding might be explained by the fact that this item was quite long and was formulated with the verb in the conditional tense, which is not frequently used in Portuguese, thereby making the meaning of the sentence more difficult to understand.

Internal consistency was calculated using Cronbach’s alpha coefficient and a satisfactory value was found, suggesting that the Portuguese version of the scale is a reliable measure. However, our results were lower than the findings in the study of the original version of the ICE-FIBQ scale (Gisladottir & Svavarsdottir, 2016; Cronbach’s α = 0.78), as well as in the study of the version developed for health care professionals, Iceland Health Care Practitioner Illness Beliefs Questionnaire (ICE-HCP-IBQ; Svavarsdottir et al., 2018; Cronbach’s α = 0.92), and the Spanish version of ICE-HCP-IBQ (Alfaro-Diaz et al., 2020; Cronbach’s α = 0.83).

In addition, a test–retest reliability analysis calculated through ICC showed a satisfactory stability over time (0.71), approximately 4 weeks following the first administration, meaning that illness beliefs, as expected, remain stable over time. Concordantly, the authors of the Spanish version of ICE-HCP-IBQ (Alfaro-Diaz et al., 2020) found similar results (0.72).

Validity was assessed through construct validity and the results found in CFA corroborate the seven-item single-factor model of the original version (Gisladottir & Svavarsdottir, 2016). Our findings were also similar to the structural analysis of the version developed for health care professionals, the ICE-HCP-IBQ scale, and by the Spanish version of ICE-HCP-IBQ (Alfaro-Diaz et al., 2020). Factor loadings showed that all items played an important role in the description of the construct. Contrary to most fit indices obtained, the RMSEA obtained was not the expected one; essentially, its upper limit exceeded the recommended value. It is known that this index is influenced by the sample size and by models with few degrees of freedom (Shi et al., 2019); as is the case, this index tends to obtain critical values, so we must take these aspects into account. When analyzing the modification rates, we noticed that the errors in Items 1 and 2 were correlated, and there may be some redundancy between the content of these items. However, we consider that in future studies, it would be important to analyze whether this finding persists.

As mentioned above, this instrument was developed based on the IBM (Wright & Bell, 2021) which is a conceptual framework for advanced family nursing practice with decades of research in Canada. The results in this study suggest that the instrument measures the proposed construct. The instrument under study was developed in Iceland to measure illness beliefs when offering advanced family nursing practice, but it is relevant to note that this approach of family nursing advanced practice is not common in Portugal within pediatric settings. Nonetheless, the instrument is an important and promising tool for intervention research, as it has been found to capture change following a family-level nursing intervention in a clinical population (Gisladottir & Svavarsdottir, 2016).

There were several limitations in this study. First, our sample was quite heterogeneous as it included parents with children/adolescents with a large diversity of health chronic conditions and some of them suffered from more than one illness. It would be important to continue to assess the psychometric properties of this instrument with family members who are experiencing a specific chronic disease. Second, the number of participants who responded twice to the test–retest analysis was not very large. Nevertheless, the size of this sample for the test–retest was satisfactory and showed that the ICE-FIBQ scale is an instrument with stability.

## Conclusion

The Portuguese version of ICE-FIBQ was found to be a reliable and valid instrument when used with Portuguese family members of children/adolescents with chronic health conditions. This instrument highlights the importance of nurses’ assessment of individuals’ and families’ beliefs to support and enhance family healing in the experience of pediatric chronic disease. A strength of the ICE-FIBQ is that this scale was based on the IBM that is an advanced practice model in family nursing with a solid conceptual framework that has been studied for over three decades. As such, this study contributes to improving family nursing intervention in Portugal by validating the Portuguese version of the ICE-FIBQ. The use of this instrument will be useful in addressing families’ needs and establishing effective therapeutic relationships with families. We recommend replicating this study using other samples to expand its use.
